# Overcoming immune resistance in ovarian cancer: checkpoint inhibitors, tumor microenvironment, and translational advances

**DOI:** 10.3389/fonc.2026.1762619

**Published:** 2026-02-05

**Authors:** Song Yue, Tao Wen, Xiaozhu Liu, Juan Tang, Yue Liu, Shengxian Peng

**Affiliations:** 1 Department of Clinical Laboratory, The First Affiliated Hospital of Chengdu Medical College, Chengdu, China; 2 Department of Clinical Blood Transfusion, Sichuan Provincial People’s Hospital, School of Medicine, University of Electronic Science and Technology of China, Chengdu, China; 3 Department of Critical Care Medicine, Clinical and Research Center on Acute Lung Injury, Emergency and Critical Care Center, Beijing Shijitan Hospital, Capital Medical University, Beijing, China; 4 Scientific Research Department, First People’s Hospital of Zigong City, Zigong, China; 5 Department of Pediatrics, First People’s Hospital of Zigong City, Zigong, China

**Keywords:** biomarker, chemotherapy, immune checkpoint inhibitor, ovarian cancer, PARP inhibitor, PD-1, tumor microenvironment

## Abstract

Ovarian cancer remains one of the most lethal gynecologic malignancies, with high recurrence rates and poor prognosis, particularly in platinum-resistant cases. Immune checkpoint inhibitors (ICIs), especially those targeting PD-1/PD-L1, have demonstrated success in multiple malignancies, yet their efficacy in ovarian cancer has been limited. Monotherapy with ICIs yields low response rates, prompting extensive investigations into combination strategies with chemotherapy, PARP inhibitors, and antiangiogenic agents. Some dual or triple regimens have shown promising activity, especially in biomarker-selected populations. However, immune resistance, immunosuppressive tumor microenvironment (TME), and biomarker heterogeneity remain significant barriers. This review summarizes the latest clinical progress in ICI-based therapies for ovarian cancer, evaluates current predictive biomarkers such as PD-L1 expression, TMB, and homologous recombination deficiency (HRD), and highlights the safety and toxicity profiles of immunotherapy. We also discuss the limitations of current clinical trials and the unmet need for precise immunotherapeutic strategies. Understanding the molecular and immunologic landscape of ovarian cancer is critical for identifying patients most likely to benefit from ICIs and guiding future clinical development.

## 1 Introduction

Ovarian cancer is one of the most common and lethal gynecological malignancies (1, 2). Standard-of-care treatment consists of cytoreductive surgery followed by platinum-based chemotherapy (3). Although the incorporation of targeted therapies has modestly improved outcomes, long-term prognosis remains dismal, especially for patients with platinum-resistant or recurrent disease (4). In recent years, immunotherapy—particularly immune checkpoint inhibitors (ICIs)—has emerged as a promising therapeutic option (5–7). In several malignancies, including non-small cell lung cancer, ICIs targeting programmed death 1 (PD-1) or its ligand PD-L1 have demonstrated substantial clinical benefit (8–11).

In ovarian cancer, however, their efficacy remains modest. Clinical trials have shown that PD-1/PD-L1 inhibitors used as second-line or later treatments in recurrent ovarian cancer yielded limited improvements in overall survival (OS) (12). A principal barrier to effective immunotherapy lies in the immunosuppressive nature of the ovarian tumor microenvironment (TME) (13–16). Notably, regulatory T cells (Tregs) are frequently enriched in the ovarian TME, where they attenuate cytotoxic T cell responses and facilitate immune evasion (17). This review outlines recent advances, persistent challenges, and future directions in ovarian cancer immunotherapy, aiming to inform the development of more effective, context-specific approaches.

## 2 Mechanisms of immune exclusion and resistance to immune checkpoint inhibitors in ovarian cancer

Ovarian cancer is characterized by poor infiltration of effector T lymphocytes and immune exclusion (17). This immunologically “cold” phenotype arises from a confluence of tumor-intrinsic and microenvironmental factors. Notably, ovarian cancer cells often downregulate major histocompatibility complex class I (MHC-I) molecules, thereby evading recognition and elimination by cytotoxic T lymphocytes (18, 19). Concurrently, the tumor microenvironment impairs dendritic cell (DC) maturation and antigen-presenting function, resulting in suboptimal T cell priming and weakened adaptive immune activation (20, 21). Tumor cells and associated stromal components actively recruit immunosuppressive populations, including MDSCs and Tregs, which inhibit cytotoxic immune responses via direct cell–cell contact and cytokine-mediated suppression (22). Notably, IL-10 and TGF-β, abundantly secreted by tumor and immune cells in the TME, dampen effector T cell proliferation and skew macrophage polarization toward the immunosuppressive M2 phenotype (23, 24). Moreover, tumor-derived metabolites such as lactate and adenosine further exacerbate immune dysfunction. Many ovarian cancers harbor defects in cGAS–STING pathway, a central axis in type I interferon (IFN) induction, leading to impaired type I IFN production, defective DC recruitment and maturation, and insufficient activation of adaptive immunity (25, 26). In the absence of STING signaling, DCs fail to mature effectively, compromising antigen presentation and CD8^+^ T cell priming. This cascade ultimately results in reduced CTL infiltration and reinforces tumor immune evasion (27, 28).

Immune checkpoints are regulatory molecules expressed on immune cells that modulate immune activation. Among them, PD-1, PD-L1, and cytotoxic T-lymphocyte antigen 4 (CTLA-4) have been most extensively characterized (29–31). Dysregulated expression of these molecules contributes to tumor progression and immune evasion (32). Immune checkpoint inhibitors restore antitumor immunity by interrupting these inhibitory pathways (33). Specifically, PD-1/PD-L1 inhibitors block the interaction between PD-1 on T cells and PD-L1 on tumor cells, thereby reinvigorating T cell–mediated cytotoxicity. In ovarian cancer, PD-1/PD-L1 blockade has been primarily explored in the recurrent setting (34–36). However, the efficacy of ICI monotherapy has been limited, with overall response rates (ORRs) typically approximating 10% (34). In the phase II KEYNOTE-100 trial, which enrolled 376 patients with recurrent ovarian cancer, pembrolizumab monotherapy yielded a median OS of 19 months. Higher response rates were observed in patients with elevated PD-L1 combined positive scores (CPS), although no definitive correlation between CPS and clinical outcome was confirmed (37). Notably, a phase III randomized trial involving platinum-resistant ovarian cancer patients demonstrated that nivolumab monotherapy significantly prolonged progression-free survival (PFS) compared to chemotherapy, highlighting its potential benefit in this therapeutically challenging population (38).

## 3 Combination of ICIs and anti-tumor drugs

### 3.1 Combination of ICIs with other ICIs

Monotherapy with ICIs has yielded modest efficacy in ovarian cancer, prompting investigation into “dual ICI therapy” (39, 40). The NRG GY003 trial (41) evaluated nivolumab plus ipilimumab in 100 patients with recurrent ovarian cancer following one to three prior lines of platinum-based chemotherapy. Compared to nivolumab monotherapy, the combination of nivolumab and ipilimumab significantly improved ORR (42). CTLA-4 and PD-1 modulate distinct phases of T cell activity. CTLA-4 blockade enhances priming and expansion of naïve T cells, while PD-1 inhibition reactivates exhausted effector T cells within the tumor microenvironment (43–45). This complementary interplay forms the rationale for combined blockade. However, in ovarian cancer, this synergy is muted, likely due to its immunologically “cold” microenvironment—characterized by sparse T cell infiltration, limited dendritic cell maturation, and low neoantigen burden (46). These features impair antigen presentation and effector T cell activation, thereby attenuating the therapeutic impact of dual checkpoint blockade. Similarly, neither concurrent nor sequential administration of anti-CTLA-4 and anti-PD-1 antibodies demonstrated significant progression-free survival (PFS) benefits over PD-1 monotherapy (47). In the monotherapy cohort, 32% of patients achieved stable disease (SD), while the dual-ICI arm yielded partial responses (PR) in 9% of patients. Expanding on this approach, the phase II KOGC3045 trial assessed nivolumab combined with chemotherapy in platinum-resistant or refractory ovarian cancer (48). The combination yielded an ORR of 32%, with SD in 10% and a disease control rate of 35%. Subgroup analysis revealed superior PFS in the combination arm versus paclitaxel monotherapy (**Table 1**).

**Table 1:** Immune checkpoint inhibitor monotherapy and combination strategies in ovarian cancer.

Clinical trial	Design & population	Treatment regimen	Clinical result
KEYNOTE-100	Phase II; Recurrent OC, n=376	Pembrolizumab	Higher PD-L1 CPS associated with trend toward improved response
KOGC3045	Phase II; Platinum-resistant/refractory OC	Nivolumab + Chemotherapy	Improved DCR in combo group
JAVELIN Ovarian 200	Phase III; Platinum-resistant OC, n=566	Avelumab ± PLD	No significant OS difference
LEAP-005	Phase II; Heavily pretreated OC, n=31	Lenvatinib + Pembrolizumab	Limited benefit in late-line setting
TQB2450 + Anlotinib	Phase II; Platinum-resistant/refractory OC, n=34	PD-L1 inhibitor + Anti-VEGF	Promising activity despite PD-L1 status
MEDIOLA (BRCA-mut)	Phase II; Relapsed OC, BRCA-mut, n=34	Durvalumab + Olaparib	High activity in BRCA-mutant population
TOPACIO (Niraparib + Pembro)	Phase I/II; Recurrent OC, n=62	Niraparib + Pembrolizumab	Limited efficacy overall
ATALANTE	Phase III; 1L OC	Chemo + Atezolizumab	Long-term follow-up shows no added value
IMagyn050	Phase III; Stage III–IV OC	Bev + Chemo ± Atezolizumab	Limited efficacy in both HRD^+^ and PD-L1^+^

### 3.2 Combination of ICIs with chemotherapy

Chemotherapeutic agents have been demonstrated to attenuate tumor-induced immunosuppression by suppressing Tregs and myeloid-derived suppressor cells (MDSCs), while simultaneously enhancing tumor immunogenicity through the induction of immunogenic cell death (49, 50). In a phase II multicenter trial involving 26 patients with platinum-resistant ovarian cancer, the combination of pembrolizumab and pegylated liposomal doxorubicin yielded an ORR of 26%. Similarly, nivolumab combined with pegylated liposomal doxorubicin achieved an ORR of 23% in a cohort of 40 patients with recurrent disease (51). In the larger phase III JAVELIN Ovarian 200 trial, 566 patients with platinum-resistant or refractory ovarian cancer were randomized to receive either pegylated liposomal doxorubicin alone or in combination with the anti–PD-L1 antibody avelumab (52). The combination arm demonstrated a higher ORR and a modest improvement in PFS, although OS did not differ significantly.

### 3.3 ICIs combined with PARP inhibitors

Poly (ADP-ribose) polymerase (PARP) inhibitors not only disrupt DNA repair in tumor cells but also upregulate PD-L1 expression, thereby enhancing tumor immunogenicity. The strategy of co-administering PARP inhibitors and ICIs is under active clinical investigation in ovarian cancer. In the MEDIOLA trial, olaparib plus durvalumab demonstrated a 72% objective response rate (ORR) in relapsed, platinum-sensitive ovarian cancer patients naïve to PARP inhibitors, with a heightened ORR of 81% among BRCA1/2-mutated individuals (53). Similarly, the TOPACIO study reported an ORR of ~18% for the niraparib–pembrolizumab combination in 62 patients with recurrent ovarian cancer (54). By contrast, the GOG-3032 trial—assessing PARP inhibition alongside PD-1 blockade in platinum-resistant disease—achieved a modest ORR of 7% and was subsequently terminated due to limited efficacy (55). BRCA mutations and homologous recombination deficiency (HRD) compromise homologous recombination repair (HRR), resulting in the accumulation of cytosolic DNA fragments. These fragments activate the cGAS–STING pathway, triggering type I interferon and pro-inflammatory cytokine release, which in turn enhances dendritic cell maturation, antigen presentation, and CD8^+^ T cell infiltration. Additionally, HRD-induced genomic instability elevates neoantigen burden, potentially augmenting responsiveness to immune checkpoint blockade.

### 3.4 Combination of ICIs and anti-angiogenic agents

Anti-angiogenic agents can reprogram the tumor vasculature and microenvironment to augment antitumor immunity and potentiate the response to immune checkpoint blockade (56). Pathological angiogenesis in tumors not only disrupts vessel integrity but also hinders immune cell infiltration and sustains an immunosuppressive milieu. In contrast, vascular normalization facilitates immune cell trafficking and enhances effector function (57). Several clinical efforts have evaluated the therapeutic synergy of combining PD-1/PD-L1 blockade with anti-angiogenic agents in recurrent ovarian cancer (58, 59). However, the clinical efficacy of such combinations remains limited, with ORRs typically below 20%, and the success of dual blockade strategies is often molecule-dependent. For instance, the LEAP-005 trial, which evaluated lenvatinib plus pembrolizumab in heavily pretreated patients with recurrent ovarian cancer, reported an ORR of merely 10% among 31 participants who had received at least two prior lines of therapy (60). In contrast, a singe study assessing the PD-L1 inhibitor TQB2450 in combination with the multi-targeted anti-angiogenic agent anlotinib in platinum-resistant or refractory ovarian cancer achieved an ORR of 47% in 34 evaluable patients (61).

### 3.5 Combination of ICIs with anti-angiogenic agents and PARP inhibitors

Preclinical evidence indicates that anti-angiogenic therapy may reduce tumor perfusion and oxygenation, inducing hypoxia that suppresses homologous recombination (HR) repair genes such as BRCA1/2 (62). This hypoxic milieu functionally mimics BRCA mutations even in wild-type tumors, thereby fostering a state of HR deficiency. Notably, VEGF inhibition downregulates key HR mediators including RAD51 and BRCA1, impairing double-strand break repair and shifting reliance toward error-prone pathways. This synthetic vulnerability underpins the rationale for combining ICIs, anti-angiogenic agents, and PARP inhibitors in ovarian cancer. Nevertheless, clinical trials in BRCA–wild-type populations have shown limited benefit. For example, the OPAL trial by the IMagyn050 study group reported an ORR of only 20% with durvalumab, olaparib, and bevacizumab in platinum-sensitive recurrent ovarian cancer (63). A clinical trial in 40 patients with recurrent ovarian cancer showed that pembrolizumab combined with pegylated liposomal doxorubicin yielded an ORR of 48%, with a median PFS of 10 months (64). In the ATALANTE trial, newly diagnosed patients received six cycles of standard chemotherapy plus atezolizumab, followed by maintenance therapy; however, after a median follow-up of three years, no statistically significant PFS benefit was observed compared to placebo (65). Similarly, the IMagyn050 study, which included patients with stage III–IV ovarian cancer, demonstrated that the addition of atezolizumab to bevacizumab, carboplatin, and paclitaxel failed to improve PFS, even in the PD-L1–positive subgroup (66). By contrast, the DUO-O trial targeting BRCA1/2 wild-type advanced ovarian cancer revealed that a triplet maintenance regimen of durvalumab, olaparib, and bevacizumab, following standard chemotherapy plus durvalumab, conferred superior PFS over durvalumab and bevacizumab alone (67). Notably, recent data presented at ASCO 2024 involving a late-line cohort treated with ICIs showed no significant survival benefit.

## 4 Challenges in the clinical application of ICIs in ovarian cancer

### 4.1 Adverse events and management of ICIs

Immune checkpoint inhibitor therapy is associated with adverse events (AEs) in approximately 30% of patients, though the severity and frequency vary by regimen. Notably, severe AEs are observed in 0.4% of patients receiving PD-1 monotherapy and rise to 1.2% when PD-1 inhibitors are combined with CTLA-4 blockade (68). In clinical trials involving ICIs for ovarian cancer, the most commonly reported AEs include fatigue, nausea and vomiting, arthralgia, and hypothyroidism (69). Less frequent but more serious AEs include immune-related hepatitis, pneumonitis, and myocarditis. The management of ICI-related AEs in ovarian cancer generally follows protocols established in other solid tumors (70). In addition to monitoring for common toxicities such as rash, neurological symptoms, hematological abnormalities, and endocrine dysfunction, Grade 1 AEs generally warrant observation, whereas most Grade 2 toxicities, particularly cutaneous and endocrine, can be mitigated with corticosteroids and supportive care, permitting treatment continuation. In contrast, Grade 3 events necessitate temporary discontinuation and immunosuppressive therapy, while Grade 4 reactions often mandate permanent cessation. Notably, no definitive association has been established between AE incidence and therapeutic benefit in ovarian cancer.

### 4.2 Biomarkers predictive of ICIs efficacy

The human DNA mismatch repair (MMR) system safeguards genomic integrity by correcting replication-associated errors. Deficiency in this system (dMMR) leads to the accumulation of mutations and culminates in microsatellite instability-high (MSI-H) status, a molecular hallmark that enhances tumor immunogenicity via increased neoantigen generation and subsequent immune surveillance activation (71). Tumor mutational burden (TMB), defined as the total number of somatic mutations within tumor genomes, serves as a surrogate for neoantigen load. Tumors exhibiting high TMB (TMB-H) are thus theoretically more responsive to immune checkpoint blockade. Supporting this, pembrolizumab monotherapy has been approved under a tumor-agnostic indication for patients with recurrent ovarian cancer harboring dMMR/MSI-H or TMB-H, as demonstrated in the KEYNOTE-158 trial (66). Homologous recombination deficiency (HRD)—typically resulting from BRCA mutations or defects in DNA repair pathways—represents a validated biomarker for sensitivity to PARP inhibitors and platinum-based chemotherapy in ovarian cancer (72, 73). However, its predictive value for immunotherapy efficacy remains unresolved. Subgroup analyses from immune checkpoint inhibitor monotherapy trials in ovarian cancer reveal no consistent association between BRCA mutational status and clinical benefit (74, 75). Notably, tumors harboring BRCA mutations but lacking HRD signatures exhibit reduced responsiveness to both PARP inhibition and immunotherapy. PD-L1 expression, though widely employed as a predictive biomarker across malignancies, demonstrates inconsistent prognostic utility in ovarian cancer (**Table 2**).

**Table 2:** Mechanisms of immune resistance in ovarian cancer and corresponding translational strategies.

Mechanism of immune resistance	Molecular basis	Clinical impact	Translational strategies
Low TMB/MSI-H frequency	<3% MSI-H; <5% TMB ≥10 mut/Mb in OC	Poor ICI response; limited neoantigens	Focus on TMB/MSI-H subsets (dMMR, endometrioid)
Deficient CD8^+^ T cell infiltration	Immunologically “cold” TME; lack of immune cell recruitment	Low ICI efficacy; immune exclusion	Combine ICIs with ICD-inducing chemo/anti-VEGF agents
cGAS–STING pathway inactivation	Downregulated STING signaling in OC	Impaired IFN-I response, low dendritic cell maturation	Use of STING agonists or epigenetic modulators
Treg/MDSC immunosuppression	Enriched in OC TME; suppress effector T cell function	Promotes immune escape	Target Tregs (low-dose cyclophosphamide) or MDSCs
Tumor burden & immune exhaustion	High tumor volume linked to T cell dysfunction	Decreased ICI efficacy in stage IV OC	Treat early-stage OC or debulk prior to ICI
HRD–/BRCA-wildtype status	HR-proficient tumors resist PARPi–ICI synergy	Suboptimal outcomes in OPAL trial	Artificial HRD induction via anti-VEGF hypoxia
Trial population selection bias	Recurrent/platinum-resistant cases dominate trials	Underestimates ICI benefit	Design trials for early-stage, R0 resected cases

In the IMagyn050 trial (18), 24% of enrolled patients harbored BRCA1/2 mutations and 46% were homologous recombination deficiency (HRD)-positive. However, the addition of atezolizumab to standard therapy failed to confer a significant progression-free survival (PFS) advantage in the HRD-positive subgroup. Although PD-L1 expression has been suggested as a predictive biomarker, its utility in ovarian cancer remains controversial (44, 45). For instance, one study found that PD-L1 positivity in tumor-infiltrating immune cells was not associated with improved prognosis, nor did PD-L1 status correlate with clinical outcomes in epithelial ovarian cancer (46). Nevertheless, PD-L1 may retain some prognostic significance. In the KEYNOTE-100 trial (66), a positive correlation was observed between PD-L1 expression levels and response to pembrolizumab. Similarly, in IMagyn050 (47), although no significant PFS improvement was detected in patients with PD-L1 immune cell expression ≥1%, exploratory analyses revealed that individuals with expression ≥5% derived greater benefit from combined immunotherapy. Furthermore, when PD-L1 expression was assessed using tumor cell scoring, patients with elevated PD-L1 in tumor cells experienced statistically significant benefit from immunotherapy (76). These findings underscore that the prognostic and predictive value of PD-L1 in ovarian cancer is complex, varying with both the scoring methodology and threshold used. Overall, the identification of robust and reproducible biomarkers for immunotherapy responsiveness in ovarian cancer remains a critical unmet need and warrants continued investigation.

### 4.3 Limitations of immune checkpoint inhibitor application in ovarian cancer

The variable efficacy of ICIs in ovarian cancer may be attributed to intrinsic resistance mechanisms. Even in patients initially responsive to ICIs, tumor tissues may eventually acquire resistance during treatment. The recognized resistance-related factors include defective T cell infiltration and immune exclusion, as well as low MSI-H or TMB levels (77). According to biomarker subgroup analysis from the IMagyn050 trial (78), only 3% of ovarian cancer patients exhibited TMB ≥10 mutations/Mb, and merely 0.3% were MSI-H positive, indicating generally low PD-L1 expression levels in ovarian cancer tissues. Increasing tumor burden has been recognized as a negative prognostic factor in ICI therapy. Studies have shown that across multiple tumor types (including ovarian cancer), patients with higher tumor burden, liver metastases, or advanced-stage disease respond less favorably to immunotherapy. In the IMagyn050 trial, subgroup analysis revealed that patients with stage III ovarian cancer benefited from the addition of atezolizumab, whereas no benefit was observed in stage IV patients (79). Most immunotherapy trials in ovarian cancer have been conducted in recurrent or platinum-resistant disease populations, with high tumor burden, advanced stage, and poor prognosis (80). Very few studies focus on patients with R0 resection or early-stage disease, potentially underestimating the clinical benefit of ICIs (81). Additionally, ICIs may show higher efficacy in tumors of endometrial origin, such as ovarian endometrioid carcinoma and clear cell carcinoma, which are characterized by higher rates of mismatch repair deficiency and microsatellite instability, and thus more likely to respond to immunotherapy (82).

## 5 Conclusion

ICIs represent a promising yet still developing therapeutic avenue in the management of ovarian cancer. While monotherapy has yielded limited efficacy, especially in heavily pretreated or platinum-resistant patients, combination strategies have shown greater potential. Dual checkpoint blockade, ICIs combined with chemotherapy, PARP inhibitors, or antiangiogenic agents, and even triple therapies are under active exploration. Certain biomarker-defined subsets—such as patients with MSI-H/dMMR, high TMB, or BRCA mutations—may derive more benefit from immunotherapy, though these populations remain rare. The tumor immune microenvironment in ovarian cancer is often “cold,” with low T-cell infiltration and high immunosuppressive cell activity, which further limits response. Additionally, PD-L1 expression, though widely studied, offers inconsistent predictive value depending on detection methods and scoring systems.

Moving forward, several challenges must be addressed to fully integrate ICIs into standard ovarian cancer treatment. First, robust and reproducible biomarkers are urgently needed to identify responsive patients and guide therapeutic decisions. Second, the design of clinical trials must evolve to include patients with lower tumor burden or earlier-stage disease, who may benefit more from immunotherapy. Furthermore, a deeper understanding of the immune landscape, including mechanisms of resistance such as cGAS-STING defects and immune exhaustion, is crucial. Integrating omics-based profiling, spatial immunology, and single-cell technologies may help delineate new therapeutic targets. To accelerate clinical translation, future trials should prioritize HRD-positive and MSI-H ovarian cancer subtypes that are more likely to respond to ICIs, explore synergistic regimens combining ICIs with epigenetic modifiers, and leverage spatial transcriptomic profiling to uncover immune niches and enable precision immunotherapy. Ultimately, the success of ICIs in ovarian cancer will depend on precise patient stratification, rational combination regimens, and overcoming tumor-intrinsic immune resistance mechanisms.
